# Supplementary material for: Commercial arbuscular mycorrhizal fungal inoculant failed to establish in a vineyard despite priority advantage
Source: PeerJ. 2021 Apr 22;9:e11119. doi: 10.7717/peerj.11119 (PMC8071076; doi:10.7717/peerj.11119)
Supplement: Supplemental Information 1 [file peerj-09-11119-s001.docx]

**Supplementary Material**

**Table S1 –** Plant species used in the interrow survey of inoculant establishment on off-target vegetation.

^1^Douglas et al., 1999; ^2^Turkington and Cavers, 1979; ^3^Small, 1996; ^4^Weaver and Riley, 1982; ^5^Abaye, 2018; ^6^Weaver, 2001; ^7^Francis et al., 2012; ^8^Douglas et al, 1985 ^9^Douglas et al., 2001; ^10^Hyslop et al., 1999.

| Plant Species | Root Morphology | Life Cycle | Clonal propagation |
| --- | --- | --- | --- |
| *Trifolium pratense* | taproot^1^ | perennial^1^ | Some stolons^10^ |
| *Medicago lupulina* | taproot^2^ | annual/biennial/  short-lived perennial^2^ | NA^2^ |
| *Medicago sativa* | creeping^3^ | perennial^1^ | Creeping roots^3^ |
| *Convolvulus arvensis* | rhizome^4^ | perennial^4^ | rhizome^4^ |
| *Malva neglecta* | taproot^5^ | annual/biennial^5^ | NA^5^ |
| *Lactua serriola* | fibrous^5^ | biennial/winter annual^5^ | NA^5^ |
| *Conyza canadensis* | taproot^6^ | annual^6^ | NA^6^ |
| *Erodium cicutarium* | taproot^7^ | annual^7^ | NA^7^ |
| *Setaria viridis* | fibrous^8^ | annual^8^ | NA^8^ |
| *Bromus carinatus* | fibrous^9^ | annual/biennial^9^ | NA |

## ddPCR supplementary information

### Primer and probe specificity

The primers used in this study are from Kokkoris et al. 2019 and are modified from Badri et al. 2016 with a novel reverse primer, which improves technical specifications of the primer such as reduced hairpin probability and more similar primer melting temperatures. A probe was also added to the assay to increase specificity. Collectively, the assay targets a 155 bp intergenic region approximately 1 kbp upstream of the mitochondrial LSU (also referred to as the *rnl* gene). The Badri et al. primers were developed based on 14 published AM fungal mitochondrial genomes, and then were tested against 5 different *Rhizoglomus irregulare* isolates from Canada, 5 *Rhizophagus* species, and a *Glomus* species with no off-target amplification. V. Kokkoris developed the new assay based on published mitochondrial genomes, and verified specificity against pure cultures of 26 *R. irregulare* isolates and 12 AM fungal species.

After observing positive results from pre-inoculation soil samples, we verified specificity to the isolate DAOM 197198 by generating amplicons from all positive May 2013 samples. We used only the primers in standard PCR, and the resulting products were sequenced in forward and reverse by Fragment Analysis and DNA Sequencing Services (UBC Okanagan) using Sanger sequencing. It is important to note that because the probe affects what is detected, not what is amplified, there may be amplicons produced in this reaction that are not detected in the full ddPCR assay. Two distinct sequences 112-119 bp in length were returned, and although the forward and reverse runs did not overlap, they appeared to cover most of the amplicon length. These sequences were blasted against the NCBI database with a low gap penalty to allow for the expected sequencing gap, and the results are shown in Figure S1. One sequence aligned exactly to 197198, except for the sequencing gap, while the other sequence did not align to any known isolate. This second sequence, while capable of being amplified by our primers, contained two mismatches and a single base pair deletion in the centre of the probe-binding region. The deletion would strongly impair the probe-binding probability as it would cause a frame-shift and inhibit the binding of the remaining downstream probe sequence. It is still possible, however, that probe binding to this off-target sequence would occasionally occur. Sporadic probe binding would result in a lower final fluorescence than an optimal match, and would be observed as “rain” of an intermediate fluorescence. To account for this, we set a high threshold in my post-run ddPCR analysis (Fig S2), which excludes low-fluorescence droplets from estimates of isolate abundance.

**Table S2** – Primer and probe sequences, and key statistics. The probe sequence also indicates the modifications, including a Freedom 6FAM fluorophore, an interal Zen quencher, and 3’ Iowa Black FQ quencher. T_m_ is the temperature at which the oligo will separate from its complementary target sequence. For primers, these values should be as close as possible for optimal amplification, with a T_m_ difference of two degrees or less between the two primers considered acceptable. Hairpin score quantifies the likelihood and degree of hairpin formation by an oligo, with 0 representing no predicted hairpin formation. Self-complementarity scores the degree to which primers are expected to bind to themselves, with less than 8.00 considered acceptable

| Oligo | Sequence (5’ – 3’) | Template | Length (bp) | T_m_ | Hairpin score | Self-complementarity |
| --- | --- | --- | --- | --- | --- | --- |
| Forward | AGCAAATCTAAGTTCCTCAGAG | Plus | 22 | 55.55 | 0.00 | 5.00 |
| Reverse | ACTTCTATGGCTTTGTACAGG | Minus | 21 | 55.31 | 0.00 | 6.00 |
| Probe | FAM/CCCACCAGG/ZEN/GCAGATTAATCTTCCTT/3IABkFQ | Plus | 26 | 59.50 | 0.00 | 0.00 |


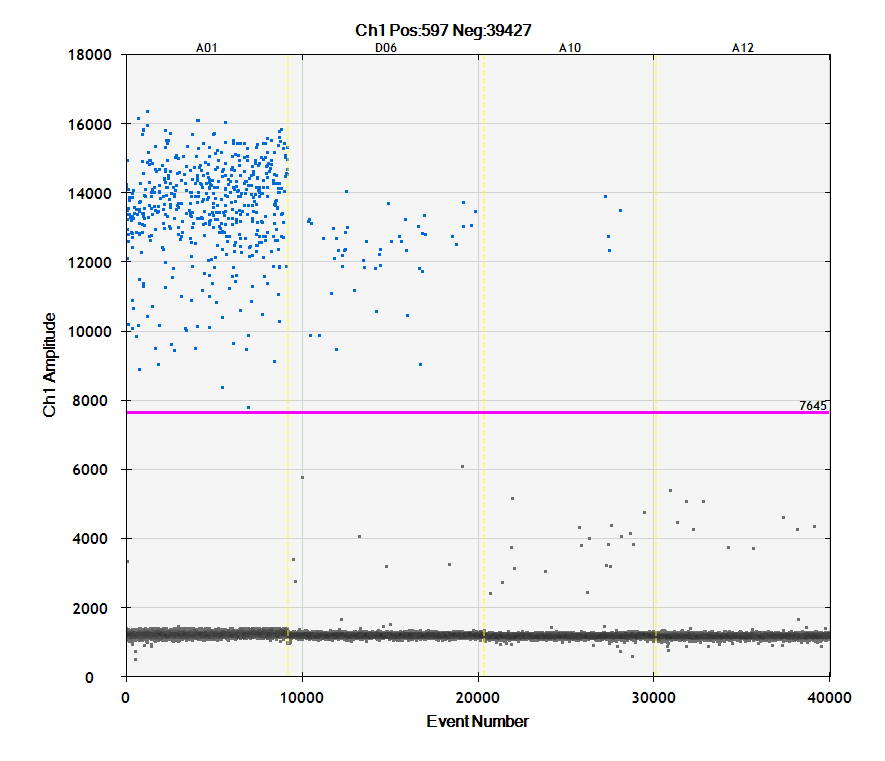


**Figure S1** – An example of ddPCR results from soil samples and the threshold that was set. A01 shows the positive control, which received amplicon produced using the primer pair as template for this reaction. Wells D06, A10, and A12 received DNA extracted from soil samples, and are examples of a moderately positive, weakly positive, and a negative sample. Droplets that produce fluorescence less than the threshold indicated by the purple line are not counted in the software’s calculation of abundance. These droplets are typically considered errors and were thus excluded.


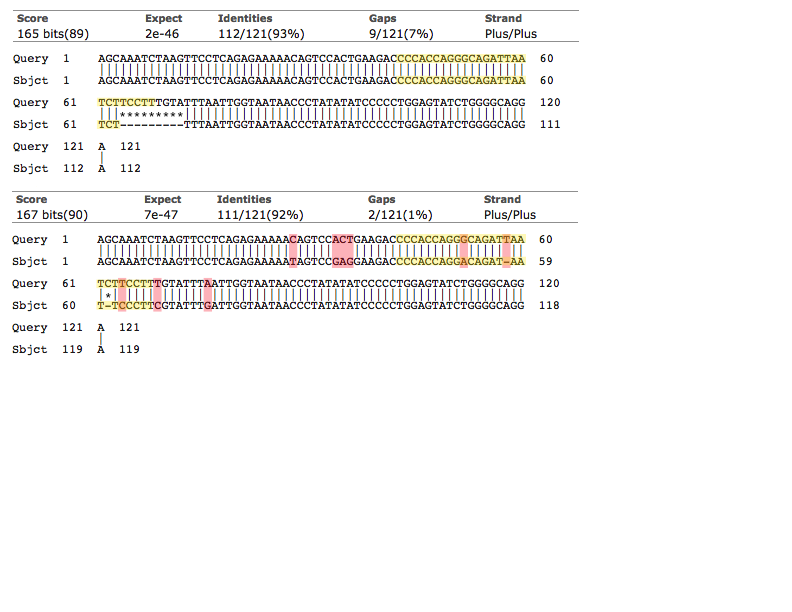


**Figure S2** – Results from local alignment of the sequences obtained from May 2013 soil samples against the target sequence from the *R. irregulare* DAOM 197198 mitochondrial large subunit rRNA. Yellow highlighting indicates probe sequence, red highlighting indicates mismatches, and asterisks denote gaps due to lack of sequencing coverage. Only two samples could be sequenced and produced two different sequences. The top sequence showed complete homology with our target sequence except for a 9 base pair sequencing gap and confirms the presence of *R. irregulare* DAOM 197198 prior to inoculation with a high degree of certainty. The second sequence contains 5 mismatch regions totalling 8 base pairs, including a single base pair deletion in the probe-binding region. While our primer set can still amplify this sequence, the mismatches and in particular the frameshift mutation would severely impair probe binding, which is the fluorescence-producing step in my assay. However, some probe binding may still occur, which could contribute to the droplets with weak fluorescence in some of my samples and supports the decision to use a high (conservative) threshold.

**Fungal abundance and spatial autocorrelation**

**Table S3** – Results of the soil chemical analyses from October 2013.

| Sample ID | P (mg/kg) | N (%) | pH | Sample ID | P (mg/kg) | N (%) | pH |
| --- | --- | --- | --- | --- | --- | --- | --- |
| E3 | 348.49 | 0.17 | 6.59 | C25 | 402.26 | 0.34 | 6.72 |
| E7 | 284.43 | 0.04 | 6.76 | C29 | 350.56 | 0.40 | 7.35 |
| E9 | 229.06 | 0.10 | 6.78 | C34 | 192.70 | 0.23 | 7.14 |
| E10 | 233.42 | 0.00 | 6.85 | C35 | 208.63 | 0.23 | 7.08 |
| E12 | 320.58 | 0.17 | 6.72 | C45 | 229.59 | 0.26 | 7.06 |
| E13 | 274.27 | 0.19 | 6.24 | C49 | 278.14 | 0.24 | 6.98 |
| E16 | 389.02 | 0.00 | 6.92 | C51 | 286.62 | 0.04 | 7.01 |
| E20 | 548.60 | 0.47 | 7.10 | C58 | 293.17 | 0.07 | 7.22 |
| E21 | 421.61 | 0.37 | 6.64 | C83 | 250.86 | 0.15 | 7.13 |
| E22 | 352.58 | 0.35 | 6.77 | C91 | 361.93 | 0.21 | 7.17 |
| E24 | 355.30 | 0.01 | 7.04 | P5 | 358.29 | 0.26 | 7.22 |
| E27 | 383.41 | 0.42 | 7.05 | P8 | 392.56 | 0.37 | 7.11 |
| E28 | 317.07 | 0.47 | 7.24 | P11 | 339.70 | 0.26 | 7.00 |
| E30 | 345.39 | 0.39 | 7.19 | P15 | 363.40 | 0.32 | 6.80 |
| E34 | 324.34 | 0.45 | 6.94 | P20 | 369.07 | 0.00 | 6.98 |
| E35 | 357.19 | 0.42 | 6.92 | P39 | 270.42 | 0.33 | 7.24 |
| E36 | 276.19 | 0.43 | 7.00 | P41 | 227.93 | 0.00 | 7.26 |
| E37 | 259.66 | 0.41 | 7.03 | P52 | 184.70 | 0.23 | 7.06 |
| E42 | 357.91 | 0.43 | 6.79 | P60 | 258.85 | 0.18 | 7.15 |
| E45 | 335.04 | 0.43 | 7.15 | P61 | 320.33 | 0.33 | 7.12 |
| E47 | 278.02 | 0.29 | 7.29 | P62 | 219.37 | 0.38 | 6.75 |
| E49 | 325.19 | 0.34 | 7.13 | P63 | 356.37 | 0.33 | 7.02 |
| C1 | 277.33 | 0.30 | 6.99 | P65 | 308.70 | 0.01 | 7.07 |
| C3 | 277.01 | 0.30 | 6.97 | P69 | 320.40 | 0.24 | 7.27 |
| C4 | 400.08 | 0.39 | 7.05 | P73 | 271.26 | 0.47 | 7.09 |
| C13 | 349.90 | 0.37 | 6.96 | P84 | 283.73 | 0.31 | 6.97 |
| C21 | 446.05 | 0.39 | 6.48 |  |  |  |  |

**Table S4** – Hyphal length and number of spores in soil taken from grapevines in October 2017.

| ID | Strategy | Inoc. | Soil hyphal length (metres/gram soil) | Spore density (number/gram soil) |
| --- | --- | --- | --- | --- |
| P39 | Pre. | Yes | 1.71 | 16 |
| P52 | Pre. | No | 0.1 | 19 |
| P60 | Pre. | Yes | 0.81 | 33 |
| P62 | Pre. | No | 0.93 | 33 |
| P63 | Pre. | No | 0.46 | 11 |
| P65 | Pre. | Yes | 0.77 | 0 |
| P73 | Pre. | Yes | 1.96 | 3 |
| E27 | Est. | No | 0.65 | 27 |
| E28 | Est. | Yes | 1.05 | 41 |
| E30 | Est. | No | 0.13 | 14 |
| E36 | Est. | Yes | 1.52 | 18 |
| E37 | Est. | Yes | 1.39 | 18 |
| E47 | Est. | Yes | 0.97 | 9 |
| E49 | Est. | Yes | 1.09 | 22 |
| C21 | Co. | Yes | 0.99 | 0 |
| C4 | Co. | Yes | 1.75 | 23 |
| C43 | Co. | No | 0.9 | 20 |
| C45 | Co. | Yes | 1.04 | 0 |
| C49 | Co. | Yes | 0.92 | 8 |
| C51 | Co. | No | 0.87 | 39 |
| C58 | Co. | Yes | 0.68 | 47 |

**Table S5** – Results of the root samples taken from grapevine in October 2017. Total colonization is shown, with colonization then categorized by hyphal, arbuscular, or vesicular.

| ID | Strategy | Inoc. | Total | Hyphal | Arbusc. | Vesic. |
| --- | --- | --- | --- | --- | --- | --- |
| P65 | Pre | Y | 55 | 29 | 14 | 12 |
| E20 | Est | Y | 32 | 19 | 5 | 8 |
| E49 | Est | Y | 36 | 28 | 1 | 7 |
| C13 | Co | N | 38 | 23 | 0 | 15 |
| C3 | Co | N | 22 | 10 | 5 | 7 |
| C45 | Co | Y | 77 | 39 | 8 | 30 |
| C49 | Co | Y | 51 | 34 | 0 | 17 |

**Table S6** – Moran’s autocorrelation coefficient values for each of the sampling periods, which shows the strength and direction of the correlation between abundance of the isolate and the spatial position of the vine.

|  | May 2013 | October 2013 | October 2014 | October 2015 | October 2016 | October 2017 |
| --- | --- | --- | --- | --- | --- | --- |
| Observed Moran’s *i* | -0.03 | -0.01 | -0.01 | -0.02 | 0.01 | -0.05 |
| *P-*value | 0.58 | 0.27 | 0.40 | 0.82 | 0.40 | 0.30 |
| Expected Moran’s *i* | -0.02 | -0.02 | -0.02 | -0.02 | -0.02 | -0.02 |
| Standard deviation | 0.016 | 0.003 | 0.014 | 0.019 | 0.035 | 0.029 |
| *n* | 38 | 51 | 52 | 49 | 51 | 53 |


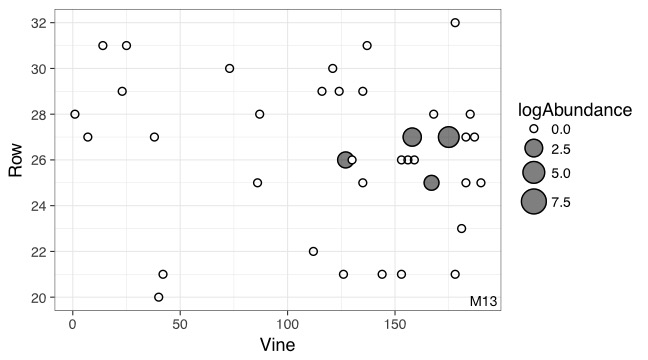


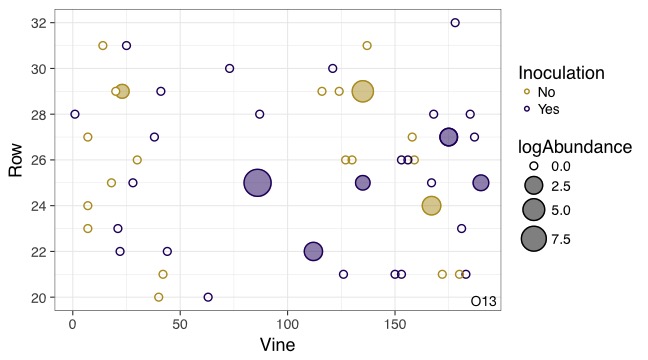


**Figures S3 (top) and S4 (bottom)** – Geographic distribution and abundance of the target isolate, *R. irregulare* DAOM 197198, in May 2013 (S3, top) and October 2013 (S4, bottom). Each point represents an individual experimental vine, and the point is filled if the sample tested positive for the presence of the isolate. The size of the point is proportional to the log abundance of the isolate. Yellow points represent non-inoculated vines, and purple points represent inoculated vines (colours omitted pre-inoculation). No statistically significant spatial autocorrelation is present.


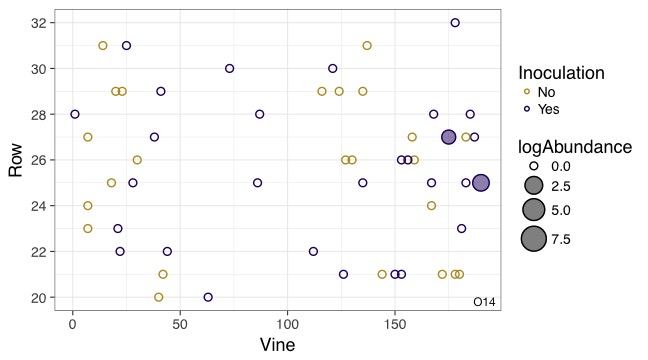


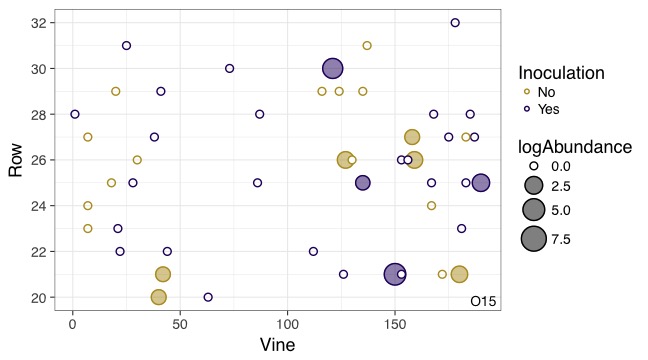


**Figures S5 (top) and S6 (bottom)** – Geographic distribution and abundance of the target isolate, *R. irregulare* DAOM 197198, in October 2014 (S5, top) and October 2015 (S6, bottom). Each point represents an individual experimental vine, and the point is filled if the sample tested positive for the presence of the isolate. The size of the point is proportional to the log abundance of the isolate. Yellow points represent non-inoculated vines, and purple points represent inoculated vines. No statistically significant spatial autocorrelation is present.


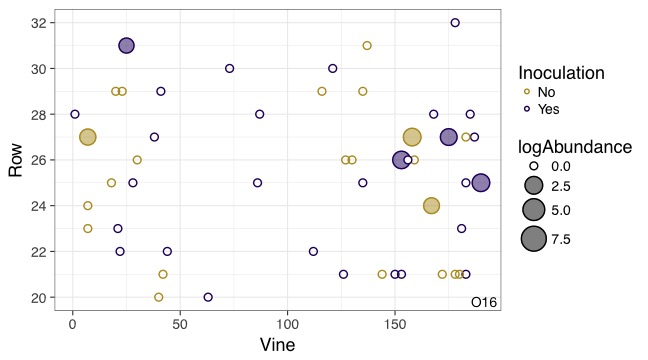


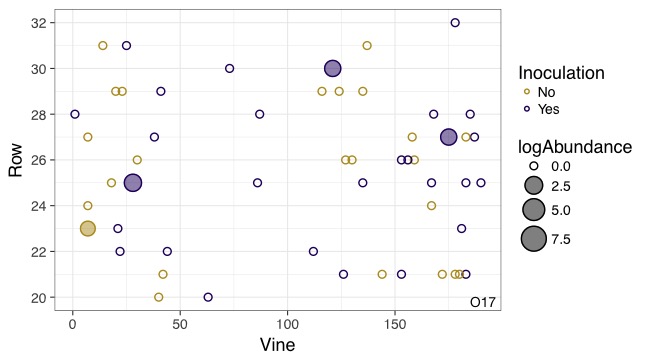


**Figures S7 (top) and S8 (bottom)** – Geographic distribution and abundance of the target isolate, *R. irregulare* DAOM 197198, in October 2016 (S7, top) and October 2017 (S8, bottom). Each point represents an individual experimental vine, and the point is filled if the sample tested positive for the presence of the isolate. The size of the point is proportional to the log abundance of the isolate. Yellow points represent non-inoculated vines, and purple points represent inoculated vines. No statistically significant spatial autocorrelation is present.

**Figure S9** - The mortality rate of all vines planted at the beginning of this experiment (*N* = 104) by October 2017. Vines inoculated with a commercial AM fungal inoculant experienced lower mortality but did not differ significantly from non-inoculated vines (Fisher’s Exact, odds ratio = 1.574, *N* = 104, *P* = 0.38).

**Figure S10** - Vine shoot length (log) did not differ between inoculated and non-inoculated vines (LMM; *X*^2^ = 0.830, *N* = 270, *P­* = 0.362). Figure shows all strategies pooled, as there was no interaction (LMM; *X*^2^ = 2.999, *N =* 270, *P­* = 0.223). In this figure and all subsequent boxplots, the box depicts the interquartile range (IQR) between the first and third quartiles, the horizontal black line represents the median, and the vertical black line represents the range up to 1.5 times the IQR. Any value falling outside this limit is represented by a black dot.

**Figure S11** - There was no effect of inoculation on the base diameter of shoots produced by inoculated and non-inoculated vines (LMM; *X*^2^ = 2.625, *N =* 190, *P­* = 0.269). Figure shows all strategies pooled, as there was no interaction effect (LMM; *X*^2^ = 0.424, *N =* 190, *P­* = 0.810).

**Figure S12** - The percentages of vines that produced at least one cluster of grapes, which did not differ between inoculated and non-inoculated vines (Fisher’s Exact, odds ratio = 0.700, *N =* 88, *P­* = 0.505). Figure shows all strategies pooled, as there was no interaction effect.

**Figure S13** - Among vines that produced grapes, inoculation did not increase the number of grape clusters produced (LMM, *X*^2^ = 0.427, *N =* 70, *P­* = 0.514). Figure shows all strategies pooled, as there was no interaction (LMM, *X*^2^ = 2.720, *N =* 70, *P­* = 0.257).


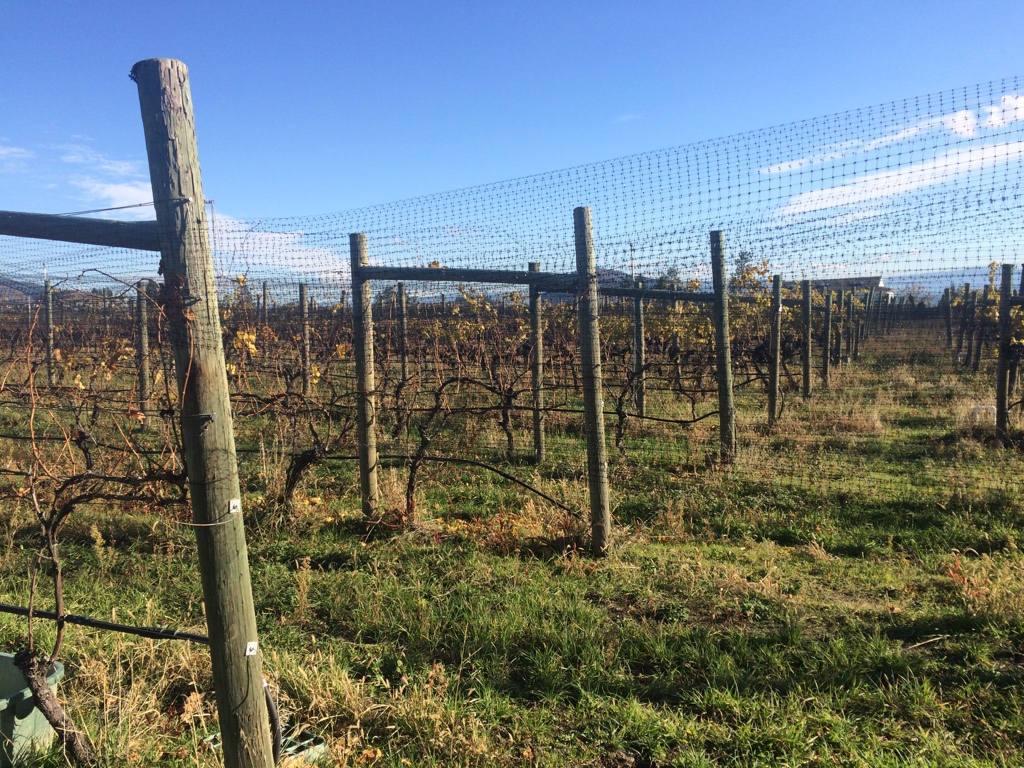


**Photo S1 –** A view of our study site across rows during an October sampling visit with bird netting in place. Photo credit: Corrina Thomsen.


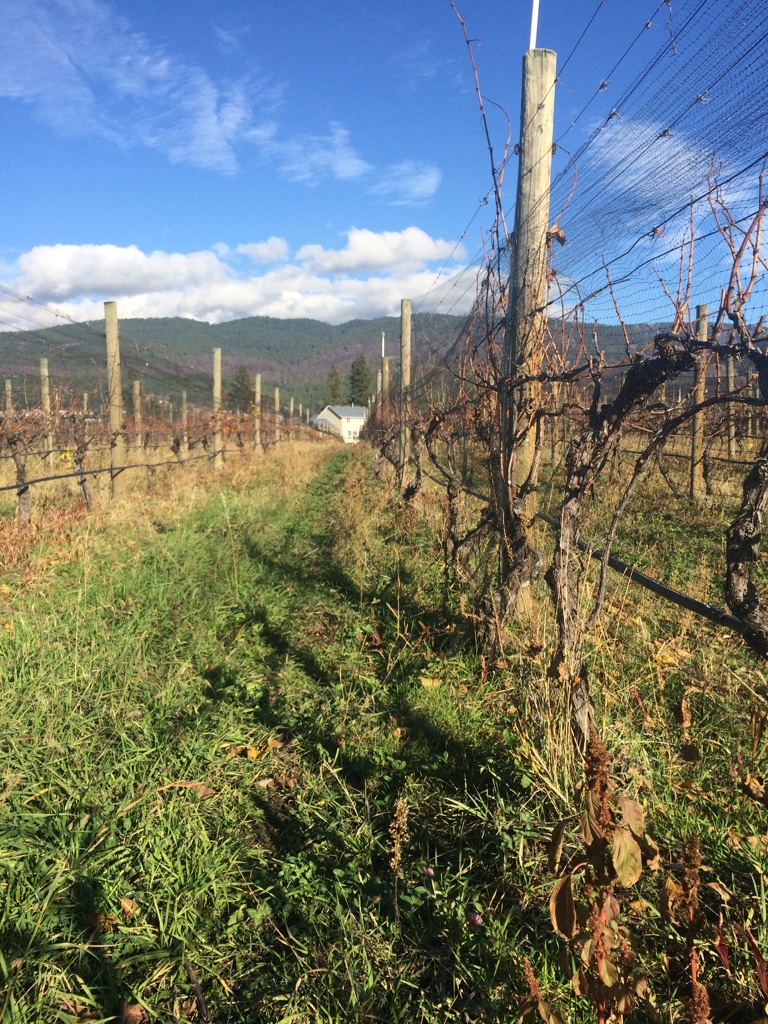


**Photo S2 –** A view down a row during an October sampling visit. Photo credit: Corrina Thomsen.


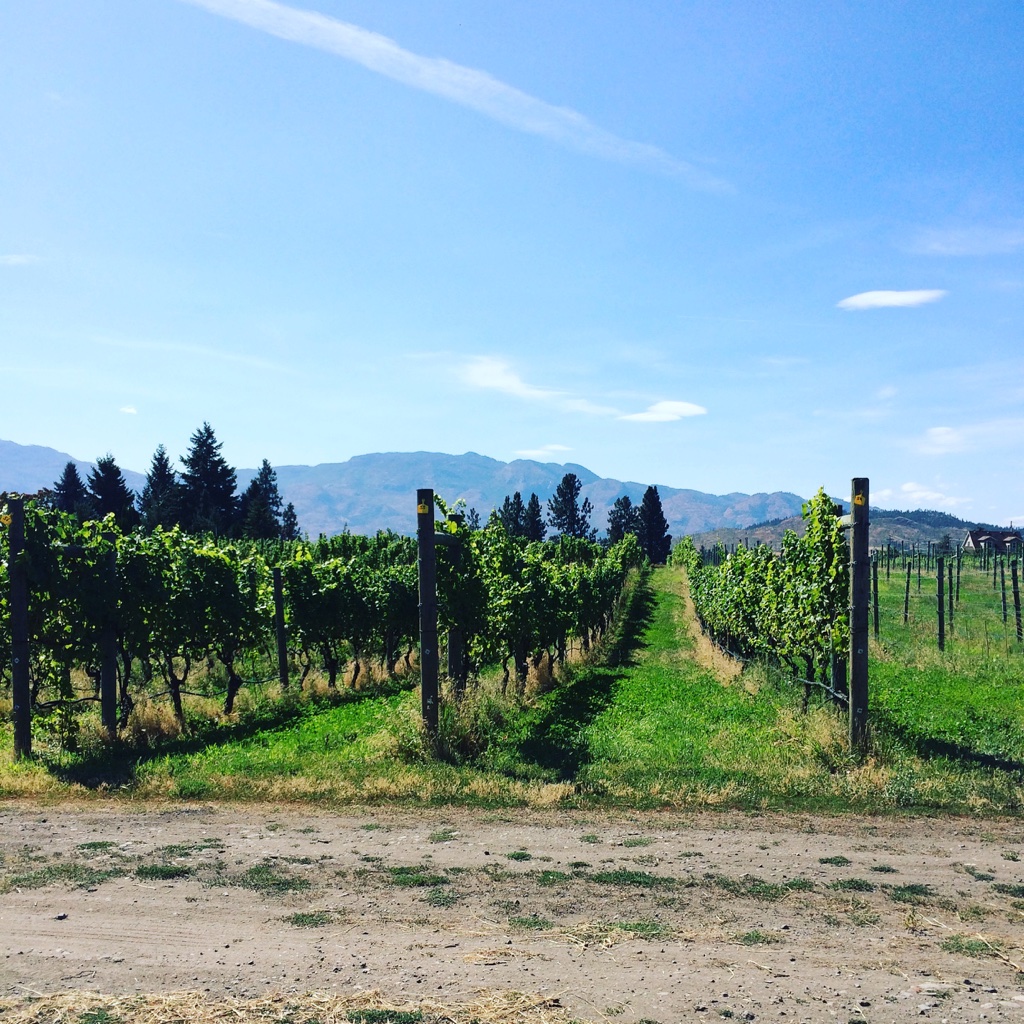


**Photo S3** - A view of the rows in early summer. Photo credit: Corrina Thomsen.


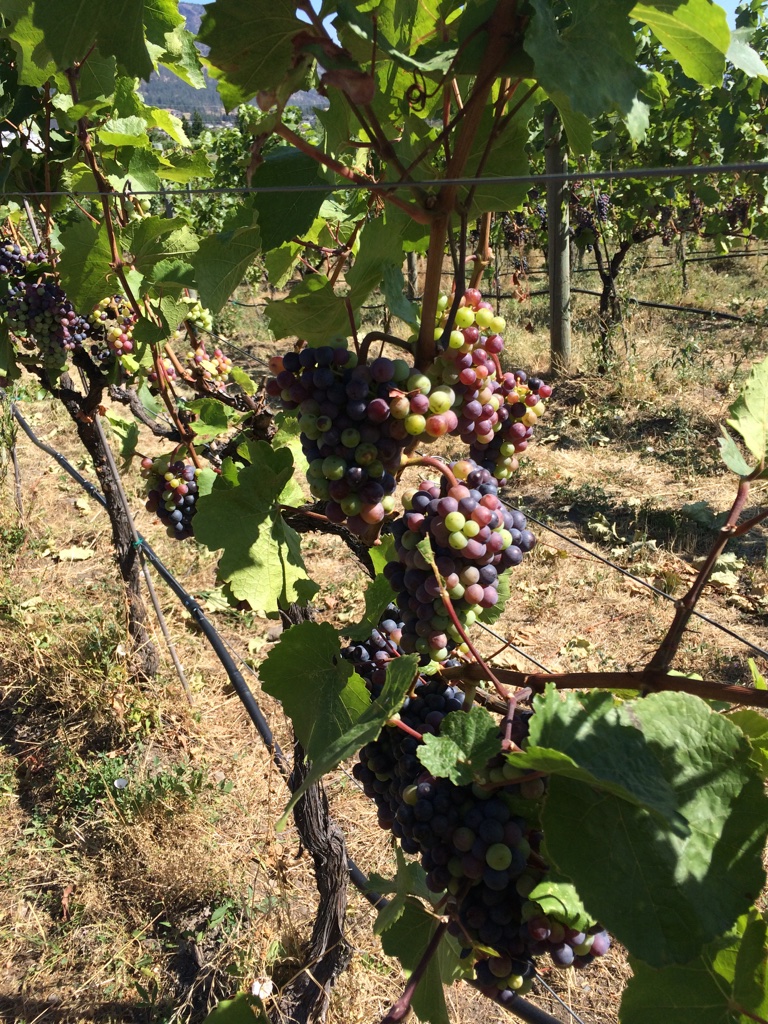


**Photo S4** – A view of individual vines in late summer. Photo credit: Corrina Thomsen.
